# Production of 2,3-butanediol in *Saccharomyces cerevisiae* by *in silico* aided metabolic engineering

**DOI:** 10.1186/1475-2859-11-68

**Published:** 2012-05-28

**Authors:** Chiam Yu Ng, Moo-young Jung, Jinwon Lee, Min-Kyu Oh

**Affiliations:** 1 Department of Chemical & Biological Engineering, Korea University, Seoul, 136-701, Republic of Korea; 2 Department of Chemical & Biomolecular Engineering, Sogang University, Seoul, 121-742, Republic of Korea

**Keywords:** 2,3-Butanediol, *Saccharomyces cerevisiae*, Metabolic engineering, Flux balance analysis, Alcohol dehydrogenase, OptKnock

## Abstract

**Background:**

2,3-Butanediol is a chemical compound of increasing interest due to its wide applications. It can be synthesized via mixed acid fermentation of pathogenic bacteria such as *Enterobacter aerogenes* and *Klebsiella oxytoca.* The non-pathogenic *Saccharomyces cerevisiae* possesses three different 2,3-butanediol biosynthetic pathways, but produces minute amount of 2,3-butanediol. Hence, we attempted to engineer *S. cerevisiae* strain to enhance 2,3-butanediol production.

**Results:**

We first identified gene deletion strategy by performing *in silico* genome-scale metabolic analysis. Based on the best *in silico* strategy, in which disruption of alcohol dehydrogenase (ADH) pathway is required, we then constructed gene deletion mutant strains and performed batch cultivation of the strains. Deletion of three ADH genes, *ADH1, ADH3* and *ADH5,* increased 2,3-butanediol production by 55-fold under microaerobic condition. However, overproduction of glycerol was observed in this triple deletion strain. Additional rational design to reduce glycerol production by *GPD2* deletion altered the carbon fluxes back to ethanol and significantly reduced 2,3-butanediol production. Deletion of *ALD6* reduced acetate production in strains lacking major ADH isozymes, but it did not favor 2,3-butanediol production. Finally, we introduced 2,3-butanediol biosynthetic pathway from *Bacillus subtilis* and *E. aerogenes* to the engineered strain and successfully increased titer and yield. Highest 2,3-butanediol titer (2.29 g·l^-1^) and yield (0.113 g·g^-1^) were achieved by *Δadh1* Δ*adh3* Δ*adh5* strain under anaerobic condition.

**Conclusions:**

With the aid of *in silico* metabolic engineering, we have successfully designed and constructed *S. cerevisiae* strains with improved 2,3-butanediol production.

## Background

With soaring oil price but indefinitely high demand for petroleum, various sustainable forms of alternative energy and chemicals have been sought after. Microorganisms are able to utilize a wide range of substrate such as plant biomass or agricultural waste and convert them into valuable chemicals and biofuel. With rapid development in microbial engineering technology, this bio-based refinery will be more feasible in terms of cost in the future and eventually reduce the dependency on fossil fuel.

**Figure 1:**
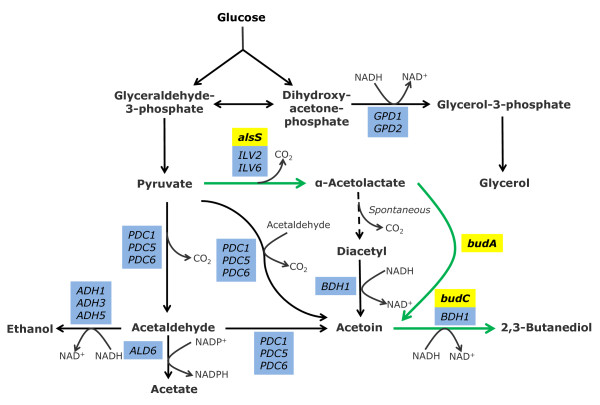
**Metabolic pathways for 2,3-butanediol biosynthesis and pyruvate metabolism in *S. cerevisiae*.** Green arrow shows the bacterial 2,3-butanediol synthesis pathway through α-acetolactate decarboxylase. Blue boxes contain the *S. cerevisiae* innate genes, while yellow boxes indicate the foreign genes introduced to the engineered strains.

2,3-Butanediol is an interesting metabolic product as its derivatives can be used in wide arrays of industries ranging from synthetic rubber, solvents and drugs. 2,3-Butanediol can be produced efficiently via mixed acid fermentation with prokaryotes such as *Klebsiella pneumoniaKlebsiella oxytocaEnterobacter aerogenesSerratia*, and *Bacillus polymyxa*[1]. In these bacteria, pyruvate is first converted into α-acetolactate by acetolactate synthase. In anoxic state, α-acetolactate decarboxylase catalyzes the conversion of α-acetolactate into acetoin (Figure 1, green arrow). In the presence of oxygen, spontaneous decarboxylation of α-acetolactate produces diacetyl. Diacetyl reductase then converts diacetyl into acetoin. 2,3-Butanediol is resulted from the reduction of acetoin by butanediol dehydrogenase.

Most of these bacteria, however, belong to class 2 microorganisms, which are not desirable in industrial-scale fermentation in terms of safety regulations [2]. The need for safe 2,3-butanediol producers are undeniably important when 2,3-butanediol are used as precursors for food additives and cosmetics. Yang et al. has successfully screened a GRAS (generally regarded as safe) microorganism *Bacillus amyloliquefaciens* B10-127 that has high yield (0.42 g·g^-1^glucose) and could produce up to 92.3 g·l^-1^ of 2,3-butanediol during fed-batch fermentation [3]. The model eukaryotic microorganism *Saccharomyces cerevisiae* which is widely used in the industry is also a promising host microorganism.

In *S. cerevisiae*, acetaldehyde, pyruvate and α-acetolactate are the precursors of 2,3-butanediol (Figure 1). The biosynthetic pathway with diacetyl as intermediate is similar with that of the bacteria’s. However, α-acetolactate decarboxylase is not found in most of the S*. cerevisiae* strain. Instead, *S. cerevisiae* can synthesize acetoin via the condensation of active acetaldehyde with acetaldehyde by pyruvate decarboxylase [4]. In addition, the carboligase mechanism of pyruvate decarboxylase for the synthesis of acetoin from the reaction between pyruvate and acetaldehyde has been elucidated previously [5]. Acetoin is then converted into 2,3-butanediol by butanediol dehydrogenase.

Hitherto, there are few reports regarding 2,3-butanediol production by engineered *S. cerevisiae* strain. 2,3-Butanediol production has been favored over acetoin and diacetyl in beer and wine fermentation due to its neutral organoleptic characteristics. Diacetyl production in brewing process is undesirable due to its unpleasant flavor and its low taste threshold. Previous attempts successfully reduced the diacetyl production in brewer’s yeast by expressing heterologous α-acetolactate decarboxylase from *Klebsiella terrigena* and *Enterobacter aerogenes*[6,7]. 2,3-Butanediol which has a higher flavor threshold than diacetyl is subsequently produced as the final product. Other than that, overexpression of *GPD1* in wine yeast strains also led to increased 2,3-butanediol production [8]. Recently, Ehsani et al. [9] have shown that by the overproduction of native Bdh1p or by changing the coenzyme specificity of *BDH1* of a strain that overproduces glycerol and lacks *ALD6*, they manage to yield 0.0307 - 0.033 g·g^-1^ of 2,3-butanediol in synthetic medium that simulated standard grape juice. Nevertheless, the focus of previous attempts did not target 2,3-butanediol as the main product.

Despite possessing more than two innate pathways that led from pyruvate to 2,3-butanediol [10], the productivity is extremely poor in wild-type *S. cerevisiae* strain when compared with bacteria[2]. We believed that the capability of *S. cerevisiae* in producing 2,3-butanediol can be further improved. For this purpose, computational strain design and optimization procedures can guide researcher in seeking for the best metabolic engineering strategy that coupled product formation with cellular objective such as growth [11]. Several genome-scale reconstruction models of yeast have been published previously [12-15]. Using these constraint-based stoichiometric models of yeast, we can predict the metabolic flux redistribution in a strain caused by a genetic or an environmental perturbation by flux balance analysis (FBA) methods [16,17].

OptKnock framework[18] was one of the earliest FBA-based algorithms being developed to predict gene deletion strategies for the overproduction of a target chemical while maximizing the cellular objective such as growth. Since then, this algorithm has been successfully applied to design *Escherichia coli* strain for the overproduction of lactate [19], malonyl-CoA [20] and 1,4-butanediol [21]. In comparison with *E. coli* stoichiometric models, the eukaryote *S. cerevisiae* models are compartmentalized and more complex in terms of its metabolic network. Constraint-based modeling approach for chemical overproduction in *S. cerevisiae* has been received with mixed opinions [22-24]. Kennedy et al. demonstrated a successful FBA-based design approach for the production of formic acid, a non-fermentative metabolite in yeast [25]. Meanwhile, Bro et al. has applied the genome-scale cell model of *S. cerevisiae* to construct a strain with 40% reduced glycerol yield and increased ethanol yield [26].

In this paper, we attempted to engineer *S. cerevisiae* strain for the production of 2,3-butanediol. To achieve this objective, *in silico* strain design was performed with a recently published *S. cerevisiae* genome-scale metabolic model of yeast, iMM904 [13]. OptKnock algorithm identified genes that have to be deleted for the overproduction of 2,3-butanediol. Based on the *in silico* prediction, we constructed a few strains with different combinations of gene deletions and quantitatively analyzed the extracellular metabolites in batch fermentations. We also evaluated whether two other strategies that would reduce by-products and increase pre-cursors of 2,3-butanediol will favor its production: 1. deletion of a NADP^ + ^dependent aldehyde dehydrogenase encoded by *ALD6* and 2. deletion of NADH dependent glycerol-3-phosphate (G3P) dehydrogenase encoded by *GPD2*. Finally, we compared the effect of the amplification of *S. cerevisiae* innate 2,3-butanediol biosynthesis pathway with the introduction of bacterial pathway to different engineered host strains.

## Results and discussion

### *In silico* design of 2,3-butanediol producing strain using OptKnock

The flux through butanediol dehydrogenase leading to the biosynthesis of R,R-2,3-butanediol was being considered as our bioengineering objective and biomass production as the cellular objective in OptKnock. All simulations were performed under aerobic condition with glucose as the substrate. We could not identify single reaction deletion or double reaction deletion strategy that will yield significant amount of 2,3-butanediol in iMM904 model.

As we proceeded with higher numbers of reactions for deletion, we initially obtained model C and model D with four and five target reactions respectively (Table 1). Both models include the deletion of pyruvate dehydrogenase (PDH) complex and glutamate dehydrogenase. The former is a mitochondrial multi-protein complex that catalyzes the conversion of pyruvate to acetyl-coA with concomitant generation of NADH [27]. With the deletion of this reaction, more flux from the pyruvate node could be redistributed towards the bioengineering objective. However, the deletion of PDH reaction reduces NADH formation that would favor 2,3-butanediol production. Other than that, the deletion of PDH complexes required deletion of up to five genes encoding different subunits that formed the complex. The NADPH dependent glutamate dehydrogenase encoded by *GDH1*and *GDH3* genes is responsible for glutamate biosynthesis and has a vital role in ammonium metabolism in yeast. It has been reported that deletion of *GDH1* increased ethanol formation and reduced glycerol formation in wild-type strain but reduced growth [28]. We could not clearly interpret the other reactions predicted in model C and D as they are non-intuitive. Aspartate transaminase is an important enzyme in amino acid metabolism that catalyzes the reversible conversion of aspartate and α-ketoglutarate to glutamate and oxaloacetate. NAD kinase is responsible for the phosphorylation of NAD^+^ to NADP^+^; but the combination of *POS5* and *UTR1* genes deletion is synthetically lethal [29]. Meanwhile, cytidylate kinase and nucleoside diphosphate kinase are reactions in nucleotide salvage pathway, whereas thymidine phosphorylase is an enzyme in purine and pyrimidine biosynthesis pathway (Additional file 1(a)).

**Table 1:** Deletion strategies suggested by OptKnock algorithm for the production of 2,3-butanediol

**ID**	Target reaction for knockouts*	**Corresponding gene(s) for knockouts**	**Maximum specific growth rate (1/hr)**	**R,R-2,3-Butanediol yield (g/g glucose)**
WT	-	-	0.288	0
A	1. ALCD2ir:	*ADH1, ADH4, ADH5*	0.231	0.313
	2. ALCD2irm	*ADH3*		
	3. ALCD2x	*SFA1*		
B	1. ALCD2ir	*ADH1, ADH4, ADH5*	0.190	0.362
	2. ALCD2x	*SFA1*		
	3. GTPCI	*FOL2*		
	4. MDH	*MDH2*		
C	1. GLUDyi	*GDH1*, *GDH3*	0.266	0.151
	2. NADK	*UTR1, UTR2, POS5*		
	3. NDPK3	*YNK1*		
	4. PDHm	*PDB1, PDA1, LAT1, LPD1*, *PDX1*		
D	1. ASPTA	*AAT2*	0.264	0.151
	2. CYTK1	-		
	3. GLUDyi	*GDH1*, *GDH3*		
	4. PDHm	*PDB1, PDA1, LAT1, LPD1, PDX1*		
	5. TMDPP	*PNP1*		

* Additional descriptions are provided in Additional file 1.

The maximum specific growth rate and R,R-2,3-butanediol yield were calculated based on 10 mmol·gDCW^-1^.hr^-1^ of glucose uptake. WT stands for the complete network of iMM904 model.

By removing these reactions from the set of target reactions for knockout, we managed to obtain model A. In this model, elimination of all alcohol dehydrogenase (ADH) reactions was proposed. 2,3-Butanediol and ethanol not only share the same precursor molecules, which are pyruvate and acetaldehyde; the conversion of these precursor molecules to 2,3-butanediol and ethanol also require the input of NADH. Thus, removal of the highly active alcoholic fermentation pathway of *S. cerevisiae* may favor the production of 2,3-butanediol by increasing the supply of pyruvate, acetaldehyde and NADH. Even though this strain has a growth rate 20% lower than that of the reference strain, it is predicted to yield 0.313 g·g^-1^ glucose of 2,3-butanediol (Additional file 1(b)).

Another quadruple deletion mutant strain with 15% higher yield of 2,3-butanediol was also proposed as a solution. In addition to the cytosolic ADH reaction of model A, cytosolic malate dehydrogenase (*MDH2*), which converts malate to oxaloacetate with a concomitant reduction of NAD^+^, was suggested as a target reaction. This gluconeogenic reaction is subject to glucose repression. However, during simulation with iMM904 model, this flux showed negative value for glucose grown wild-type strain, suggesting that the opposite reaction is active. OptKnock might have suggested the deletion of this reaction, which competes for NADH with 2,3-butanediol. The reason for GTP cyclohydrolase deletion is unknown. Furthermore, this quadruple deletion mutant has a growth rate equal to 66% of the reference strain.

Due to the large number of reactions in the genome scale metabolic model, computational strain design with OptKnock produced some non-intuitive deletion strategies that are difficult for logical interpretation. Though, OptKnock has predicted the deletion of PDH and ADH reactions, which are highly competitive for the carbon flux to 2,3-butanediol, as our strain design strategies. Since model A and model B have higher 2,3-butanediol yields than model C and model D (Table 1), we decided to evaluate the deletion of the ADH reaction that is required in both model A and model B.

### Strain construction and microaerobic fermentations

To test this hypothesis provided by OptKnock, we would have to delete all the genes that are responsible for the conversion of acetaldehyde to ethanol. A total of four ADH genes, *ADH1ADH3ADH4* and *ADH5*, along with *SFA1* gene have to be disrupted. Previous report [30] showed that *ADH4* gene product does not contribute to ethanol production. Meanwhile, *SFA1* is known as a long-chain ADH [31] and formaldehyde dehydrogenase [32]. Therefore, our target genes for deletion were reduced to three – *ADH1ADH3* and *ADH5.*

**Figure 2:**
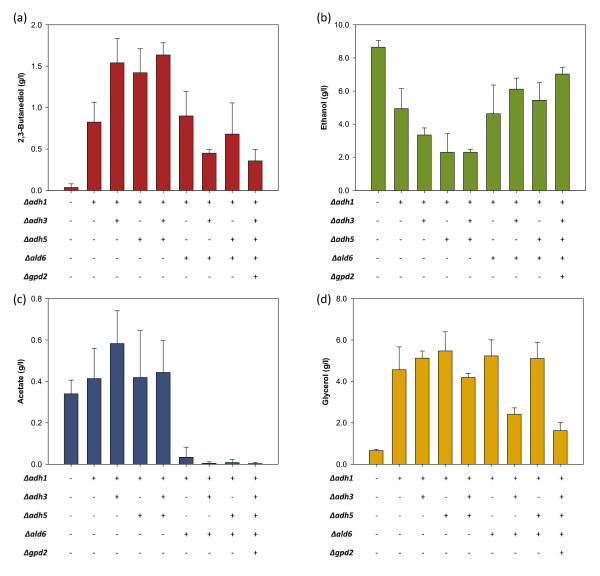
**Extracellular metabolite concentrations for batch cultivation.** Concentration of **(a)** 2,3-butanediol, **(b)** ethanol, **(c)** acetate and **(d)** glycerol at the end of batch cultivation under microaerobic conditions. Plus or minus signs indicate presence or absence of the corresponding gene deletion. The first column corresponds to the wild-type strain. Error bars represent standard deviations associated with four independent experiments (except for strains B2C-a1, B2C-a1a5a6, B2C-a1a3a5a6g2, where n = 3; and for strains B2C-a1a3a5 and B2C-a1a3a6, where n = 2).

To identify the contribution of each gene deletion to 2,3-butanediol yield, we have constructed deletion strains with different combination of the three ADH genes (Table 2). Shake-flask cultivations were then performed with these strains at microaerobic condition. Engineered strains took longer time (~ 60 h) to complete fermentation than the wild-type strain (~ 40 h). Only up to 0.04 g·l^-1^ of 2,3-butanediol (0.002 g·g^-1^) was detected in the culture of BY4742 wild-type strain. In comparison with the wild-type strain, single deletion of the major ADH gene *ADH1* reduced ethanol yield by almost half and increased 2,3-butanediol yield by up to 20.5-fold, with a 2,3-butanediol maximum titers and product yields of 0.825 g·l^-1^ and 0.041 g·g^-1^, respectively (Figure 2 and Table 3). The double deletion strains B2C-a1a3 (*Δadh1 Δadh3*) and B2C-a1a5 (*Δadh1 Δadh5*) produced up to 1.54 g·l^-1^ and 1.42 g·l^-1^ of 2,3-butanediol, respectively, with a 2,3-butanediol yield of 85% - 88% more than the *Δadh1* single deletion strain. Strain B2C-a1a3a5, of which three ADH isozymes encoded by *ADH1ADH3* and *ADH5* genes were deleted, achieved the highest 2,3-butanediol titer and yield among the engineered strains, up to 1.64 g·l^-1^ and 0.093 g·g^-1^. This triple deletion strain produced 73.3% lesser ethanol than wild-type strain. Consistent with previous reports [33,34], this strain still retains its ability to produce ethanol due to residual ADH isozymes. It has higher 2,3-butanediol yield per substrate than strain B2C-a1a3 and strain B2C-a1a5 owing to the fact that up to 2.41 g·l^-1^ of residual glucose was detected at the end of fermentation.

**Table 2:** ***S. cerevisiae*strains used in this study**

**Strain**	**Genotype**	**Reference**
BY4742	*MATα his3Δ1 leu2Δ0 lys2Δ0 ura3Δ0*	EUROSCARF
B2C-a1	BY4742 Δ*adh1::loxP*	This study
B2C-a1a3	BY4742 Δ*adh1::loxP* Δ*adh3::loxP*	This study
B2C-a1a5	BY4742 Δ*adh1::loxP* Δ*adh5::loxP*	This study
B2C-a1a3a5	BY4742 Δ*adh1::loxP* Δ*adh5::loxP* Δ*adh3::loxP*	This study
B2C-a1a6	BY4742 Δ*adh1::loxP* Δ*ald6::loxP*	This study
B2C-a1a3a6	BY4742 Δ*adh1::loxP* Δ*ald6::loxP* Δ*adh3::loxP*	This study
B2C-a1a5a6	BY4742 Δ*adh1::loxP* Δ*ald6::loxP* Δa*dh5::loxP*	This study
B2C-a1a6g2	BY4742 Δ*adh1::loxP* Δ*ald6::loxP* Δ*gpd2::loxP*	This study
B2C-a1a3a5a6g2	BY4742 Δ*adh1::loxP* Δ*ald6::loxP* Δ*gpd2::loxP* Δ*adh3::loxP* Δa*dh5::loxP*	This study
B2C-SB	BY4742 p423GPD-BDH1	This study
B2C-ABC	BY4742 p423GPD-budC pCTP-URA::TEF1p-alsS + PGK1p-budA	This study
B2C-a1a5-SB	BY4742 Δ*adh1::loxP* Δ*adh5::loxP* p423TEF-BDH1	This study
B2C-a1a5-ABC	BY4742 Δ*adh1::loxP* Δ*adh5::loxP* p423GPD-budC pCTP-URA::TEF1p-alsS + PGK1p-budA	This study
B2C-a1a6-SB	BY4742 Δ*adh1::loxP* Δ*ald6::loxP* p423GPD-BDH1	This study
B2C-a1a6-ABC	BY4742 Δ*adh1::loxP* Δ*ald6::loxP* p423GPD-budC pCTP-URA::TEF1p-alsS + PGK1p-budA	This study

*Genotype is described in the order of the corresponding genetic manipulations.

**Table 3:** Fermentation time, extracellular metabolite concentrations and yields for shake-flask culture under microaerobic condition

**Strain**	**Fermentation time, hr**	**Dry cell weight, g·l**^**-1**^	**Residual glucose, g·l**^**-1**^	**Acetaldehyde**^†^**, g·l**^**-1**^	**Yield, g·g**^**-1**^**glucose**				
					**2,3-Butanediol**	**Glycerol**	**Ethanol**	**Acetoin**	**Acetate**
BY4742	40	1.349	0	0.118	0.002	0.032	0.417	0	0.017
B2C-a1^‡^	56	0.787	0	0.663	0.041	0.225	0.243	0.016	0.02
B2C-a1a3	54	0.876	0	1.194	0.076	0.254	0.166	0.012	0.029
B2C-a1a5	72	0.634	1.80	1.106	0.079	0.295	0.123	0.010	0.024
B2C-a1a3a5^†^	54	0.900	2.41	1.316	0.093	0.238	0.131	0.008	0.025
B2C-a1a6	57	0.740	0.09	0.493	0.046	0.265	0.231	0.010	0.002
B2C-a1a3a6^†^	63	0.619	0.78	0.312	0.023	0.125	0.318	0.011	0
B2C-a1a5a6^‡^	54	0.842	0	0.263	0.033	0.247	0.259	0.011	0
B2C-a1a3a5a6g2^‡^	64	0.806	0	0.220	0.018	0.081	0.347	0.007	0

*All cultures were performed with an initial concentration of 20 g·l^-1^ of glucose under microaerobic condition. The extracellular metabolite concentrations and yields (g metabolite produced/g glucose consumed) were determined at the time of glucose exhaustion or when the strains ceased to consume glucose. Fermentation time indicates the mean time of the end of fermentation. Residual glucose indicates the amount of glucose detected in the supernatant at the end of fermentation.

** All data correspond to the mean of four independent experiments (n = 4) unless indicated. For all data, standard error of the mean is lower than 10%.

^‡^ Data correspond to the mean of three independent experiments (n = 3).

^†^ Data correspond to the mean of two independent experiments (n = 2).

Previous works stated that acetaldehyde accumulation in Δ*adh1* strain led to poor growth in glucose [34,35]. Acetaldehyde at a level higher than 0.3 g·l^-1^ inhibits cell growth [36]. We found that the concentrations of acetaldehyde in the supernatant at the end of fermentation were significantly higher in deletion strains (B2C-a1, B2C-a1a3, B2C-a1a5 and B2C-a1a3a5) than the wild-type strain (Table 3). Accordingly, the final biomass yields for these deletion strains were highly reduced. We also thought that perhaps the high acetaldehyde level in the supernatant, which is toxic to the cells, causes the cessation in glucose uptake of some of the deletion strains. In addition, deletion strains also exhibited 22- 71% higher level of acetate and 6 - 8-fold increase in glycerol level over wild-type strain. Analysis of strain lacking *ADH1* or *ADH1-4* isozymes previously has shown that glycerol is the main fermentation product for these strains, while acetaldehyde and acetate are produced in significant amounts [30]. This is because reducing the flux towards ethanol led to the accumulation of NADH and acetaldehyde. Generation of glycerol allows the strain to reoxidize excess NADH in the cytosols, while acetate formation reduces the acetaldehyde accumulated. Nevertheless, 2,3-butanediol production in these strains has not been reported. 2,3-Butanediol can be synthesized directly from pyruvate via yeast innate pathway. Production of 2,3-butanediol requires the oxidation of NADH, whereas oxidation of acetaldehyde to acetate reduced the cofactor NAD(P)^+^. Thus, accumulation of acetaldehyde in strains B2C-a1, B2C-a1a3 and B2C-a1a5 strains greatly increased the production of 2,3-butanediol. The results of additional gene deletion strategies to reduce by-products (acetate and glycerol) and their effect on 2,3-butanediol production are discussed in the next sections.

Production of 2,3-butanediol in the engineered strain mostly occurs through the pathways that are catalyzed by pyruvate decarboxylase. Deletion of *PDC1* from Δ*adh1* background was reported to reduce acetaldehyde production [37]. In our study on Δ*adh1* Δ*pdc1* strain, less than 0.60 g·l^-1^ of 2,3-butanediol was detected (unpublished observations). Deletion of *PDC1* from Δ*adh1* strain reduced the flux towards acetaldehyde and consequently decreased acetoin production. This attested that pyruvate decarboxylase enzyme is important in the production of acetaldehyde and the production of 2,3-butanediol. Nevertheless, the acetoin synthase activity of pyruvate decarboxylase enzyme has been reported to reach saturation level [5] at a very low concentration and increase only by 10% even with larger pool of acetaldehyde. So, further deletion of more ADH genes did not resulted in a more drastic increase in 2,3-butanediol production.

### The effect of *ALD6* gene deletion on 2,3-butanediol production

*ALD6* gene encodes the main cytosolic aldehyde dehydrogenase that catalyzes the conversion of acetaldehyde to acetate. Eglinton et al. has shown that acetic acid production can be reduced in glycerol overproducing strain of *S. cerevisiae* by deleting the *ALD6* gene [38]. As discussed in the previous section, strains B2C-a1, B2C-a1a3, B2C-a1a5 and B2C-a1a3a5 produced more acetate when compared to the wild-type strain. High production of acetic acid lowers the pH of the culture broth and can induce programmed cell death in *S. cerevisiae* cells [39]. So, we investigated the effect of *ALD6* deletion on the production of 2,3-butanediol in the engineered strains with deletion in one or more ADH genes.

Deletion of *ALD6* significantly reduced the production of acetate in all the engineered strains, from an average of 0.47 g·l^-1^ to undetectable level (Figure 2). However, this decrease in acetate did not correspond to the increase in 2,3-butanediol production in most of the engineered strains. Only strain B2C-a1a6 showed a slight increase in 2,3-butanediol yield when compared to its parent strain (B2C-a1), from 0.041 g·g^-1^ to 0.046 g·g^-1^. On the contrary, the 2,3-butanediol yield of B2C-a1a3a6 was equal to only 30% of strain B2C-a1a3. Similarly, strain B2C-a1a5a6 had a 58% lower 2,3-butanediol yield than strain B2C-a1a5 (Table 3).

We had initially anticipated that deletion of the aldehyde dehydrogenase gene, *ALD6,* in strains that contained one or more deleted ADH genes would lead to the accumulation of acetaldehyde. This acetaldehyde could then be converted to acetoin through pyruvate decarboxylase reaction and consequently improve 2,3-butanediol yield. In contrast to our initial hypothesis, strains B2C-a1a5a6 and B2C-a1a3a6 yielded higher levels of ethanol and lower levels of acetaldehyde than strains B2C-a1a5 and B2C-a1a3 which harbor the native *ALD6* gene. This suggests an increase in the activity of the remaining ADH isozymes of these mutant strains as a survival mechanism of the cells due to the toxicity of acetaldehyde. These residual ADH isozymes compete more efficiently for acetaldehyde than the acetoin-synthesizing pyruvate decarboxylase, which is another pathway for acetaldehyde detoxification. Similarly, Roustan et al. [40] have reported that the ADH activity of wine yeast increased when acetaldehyde was added to the stationary phase medium of wine fermentation.

### The effect of *GPD2* gene deletion on extracellular metabolite yields

In strains carrying deletion of *ADH1*, excess G3P is produced by G3P dehydrogenase from dihydroxyacetone phosphate while regenerating NAD^+^. Glycerol is then formed by the dephosphorylation reaction of glycerol-3-phosphatase. Two strains (B2C-a1a6 and B2C-a1a3a5) that produced glycerol as the major fermentation product under microaerobic condition were evaluated for the effect of the disruption of G3P dehydrogenase reaction.

**Figure 3:**
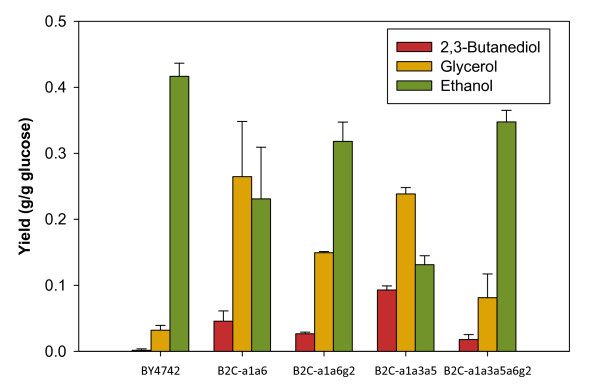
**The effect of*GPD2*deletion on 2,3-butanediol, glycerol and ethanol yields.** Metabolite yields for strains (a) BY4742, (b) B2C-a1a6, (c) B2C-a1a6g2, (d) B2C-a1a3a5 and (e) B2C-a1a3a5a6g2 at the end of batch cultivation under microaerobic condition. Columns and error bars correspond to the mean value and standard deviations of four independent experiments for (a) and (b), duplicate experiments for (c) and (d) and triplicate experiments for (e).

*GPD1* and *GPD2* encode the isozymes of G3P dehydrogenase. It has been shown that the deletion of both genes cease glycerol production in *S. cerevisiae* cells [41]. Nevertheless, deletion of both genes was lethal in strain B2C-a1a6 as the cell lost the ability to re-oxidize excess NADH and produce ATP. Single deletion of the osmoregulated *GPD1* in Δ*adh1* Δ*ald6* background has minor effect in the reduction of glycerol production (data not shown). On the contrary, the deletion of *GPD2* in strain B2C-a1a6 reduced glycerol production when compared to the double deletion strain (Figure 3). *GPD2* activity is affected by redox control. The limitation in NADH re-oxidation ability due to the deletion of *ADH1* in strain B2C-a1a6, as well as the low oxygen condition during microaerobic cultivation, contributes to the stressful condition where *GPD2* expression is highly induced. The increase in pyruvate and NADH due to *GPD2* deletion, however, did not favor 2,3-butanediol production in strain B2C-a1a6g2 as ethanol became the major fermentation product. Similarly, deletion of both *ALD6* and *GPD2* from strain B2C-a1a3a5 also completely altered the final product composition of the medium. The quintuple deletion strain displayed lower yield of 2,3-butanediol and glycerol, but higher yield of ethanol (Table 3).

In strains with *ADH1* deletion, glycerol is greatly produced to regenerate the reducing equivalents [34]. By reducing the ability of the cells to oxidize excess NADH through glycerol production, ethanol was again produced as major fermentation product in strains B2C-a1a6g2 and B2C-a1a3a5a6g2. It is possible that the expressions of the remaining ADH genes were induced to cope with the NADH surplus condition. This is because the synthesis of two moles of ethanol from one mole of pyruvate and one mole of acetaldehyde consumes twice the amount of NADH that is required for the formation of one mole of 2,3-butanediol from the same amount of precursor molecules [40].

### Anaerobic batch fermentation

**Figure 4:**
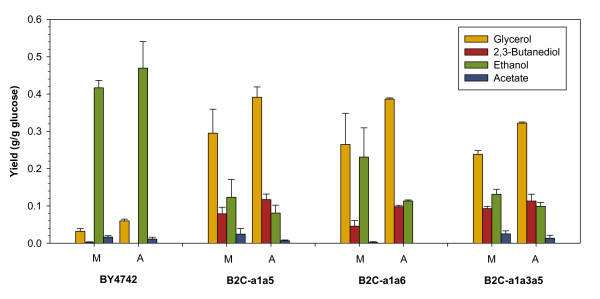
**Metabolite profile of cultivation under microaerobic (M) and anaerobic (A) conditions.** The bar chart shows glycerol, 2,3-butanediol, ethanol and acetate yields for batch fermentation of (a) BY4742, (b) B2C-a1a5, (c) B2C-a1a6 and (d) B2C-a1a3a5 under microaerobic condition and anaerobic condition with 20 g·l^-1^ of glucose as substrate. Error bars represent standard deviations.

In order to test whether 2,3-butanediol production can be improved in a highly fermentative condition, strains B2C-a1a5, B2C-a1a6, B2C-a1a3a5 and wild-type strain BY4742 were selected for anaerobic batch cultivation. In the absence of oxygen, *S. cerevisiae* cells are known to produce significant amount of ethanol and glycerol as a mean to maintain redox balance and generate ATP in the cells. Due to slower growth in anaerobic state, the biomass yield of wild-type and all engineered strains were greatly reduced while the fermentation time increased significantly. All strains exhibited higher glycerol yield due to anaerobiosis. Meanwhile, a decline in the production of the more oxidized acetate was observed in wild-type and the engineered strains (Figure 4).

**Figure 5:**
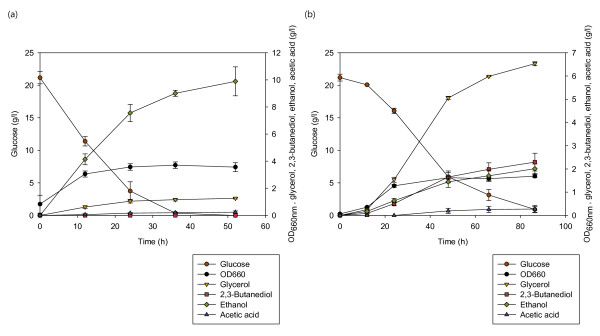
**Changes in metabolite concentrations for strains (a) BY4742 (wild-type) and (b) B2C-a1a3a5 under anaerobic condition.** Glucose (brown circles), OD_660nm_ (black circles), glycerol (inversed triangles), 2,3-butanediol (squares), ethanol (diamonds) and acetate (triangles) concentrations are shown. Symbols and error bars represent the mean and standard deviation for **(a)** triplicate and **(b)** duplicate experiments.

With deficiency in its ADH genes, the engineered strains B2C-a1a5, B2C-a1a6 and B2C-a1a3a5 produced glycerol as the major fermentation product. Under microaerobic condition, the second major by-product was ethanol, followed by 2,3-butanediol. Interestingly, growth in anoxic state led to the opposite case for strains B2C-a1a5 and B2C-a1a3a5, where 2,3-butanediol yield was higher than ethanol yield (Figure 4). In comparison to the microaerobic state, the yield of ethanol in anaerobic state was lowered by more than 34% and 24% for strains B2C-a1a5 and B2C-a1a3a5, respectively. Up to 2.29 g·l^-1^ of 2,3-butanediol was detected in the fermentation broth for the triple deletion strain, with a yield of 0.113 g·g^-1^ (Figure 5). 2,3-Butanediol was not detected in wild-type strain culture broth under anaerobic condition.

An intriguing relationship between the level of oxygen with the production concentration of 2,3-butanediol and ethanol can be deduced from our observation. 2,3-Butanediol yield correlates inversely with the level of oxygen and thus the biomass yield. As low oxygen is known to led to higher fermentative energy metabolism in *S. cerevisiae,* it is not surprising to obtain more fermentation products under anaerobic condition. It is harder to interpret the connection between 2,3-butanediol and ethanol when combined with oxygen level. Since 2,3-butanediol and ethanol shares the same precursor molecule, their concentrations should correlate in an inverse manner. We suspected that the rate of acetaldehyde formation determines the amount of ethanol and 2,3-butanediol produced. However, we could not track the dynamic changes in the concentration of intracellular acetaldehyde in this study.

### Improved 2,3-butanediol yield by gene overexpression strategies

In this section, we compared the single overexpression of innate *BDH1* and introduction of additional 2,3-butanediol synthesis pathway from bacteria in three different host: wild-type strain, strain B2C-a1a5 and strain B2C-a1a6. The heterologous pathway was introduced by the co-expression of *B. subtilis alsS* gene, *E. aerogenes budA* gene and *E. aerogenes budC* gene using two multicopy plasmids (Table 4). We used the acetolactate synthase of *B. subtilis* encoded by *alsS* gene because it has stronger preference for pyruvate over 2-ketobutyrate [42]. On the contrary, *S. cerevisiae* native acetolactate synthase encoded by *ILV2* has higher affinity for 2-ketobutyrate. *E. aerogenes budA* encodes α-acetolactate decarboxylase, which is absent in *S. cerevisiae* strain. The *E. aerogenes* 2,3-butanediol dehydrogenase, encoded by *budC*, displays higher affinity for both NADH and acetoin (K_m,NADH_ = 5–7 μM and K_m,acetoin_ = 0.4 mM) than the *S. cerevisiae* native Bdh1p (K_m,NADH_ = 45 μM and K_m,acetoin_ = 3 mM) [43,44]. The mRNA expression levels of all the genes in the strains we constructed have been confirmed by real-time PCR (data not shown).

**Table 4:** Plasmid used in this study

**Plasmid**	**Description**	**Reference**
pFA6a-KanMX6	*E. coli kan*^r^ gene under the control of *Ashbya gossypii TEF* promoter-driven	[45]
pCNKanMX6	*loxP* flanking KanMX6 at SalI/BamHI and SacI/EcoRV sites	This study
pTKURA3	*loxP* flanking *S. cerevisiae URA3* gene	This study
p423GPD	2 μ, *HIS3*, P_*GPD1*_	[46]
p423TEF	2 μ, *HIS3*, P_*TEF1*_	[46]
p423GPD-BDH1	*S. cerevisiae BDH1* gene under the control of *GPD1* promoter in p423GPD	This study
p423TEF-BDH1	*S. cerevisiae BDH1* gene under the control of *TEF1* promoter in p423GPD	This study
p423GPD-E.a.budC	*E. aerogenes* KCTC 2190 *budC* gene under the control of *GPD1* promoter in p423GPD	This study
pESC-URA	2 μ, *URA3*, P_*GAL10*_ P_*GAL1*_	Stratagene
pESC-LEU	2 μ, *LEU2*, P_*GAL10*_ P_*GAL1*_	Stratagene
pESC-LEU-Cre	P1 bacteriophage Cre recombinase under the control of *GAL10* promoter	This study
pCTP-URA	2 μ, URA3, P_*TEF1*_ P_*PGK1*_	This study
pCTP-URA::TEF1p-alsS + PGK1p-budA	*Bacillus subtilis alsS* gene under the control of *TEF1* promoter and *E. aerogenes* KCTC 2190 *budA* gene under the control of *PGK1* promoter in pCTP-URA	This study

**Figure 6:**
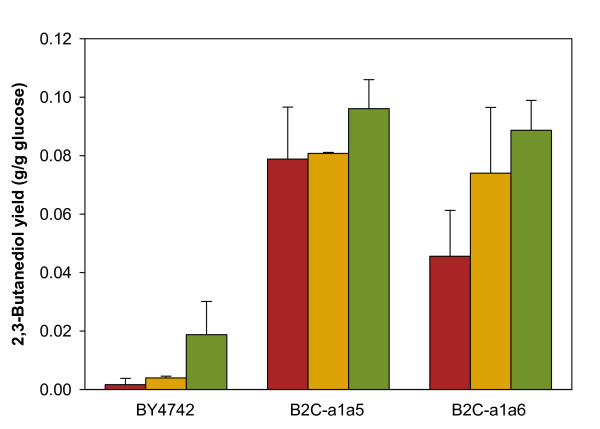
**Comparison of 2,3-butanediol yield with gene overexpression strategies.** Each bar represents the 2,3-butanediol yield for shake-flask cultivation under microaerobic condition for three different host strains: (a) BY4742, (b) B2C-a1a5 and (c) B2C-a1a6 expressing . Red bars, host strain (reference); yellow bars, overexpression of *S. cerevisiae* innate *BDH1*; green bars, bacterial *alsS-budA-budC* co-expression. Values and error bars correspond to the mean and standard deviation for triplicate experiments (except for all the red bars, where four independent experiments were conducted)

In the wild-type strain, overexpression of *BDH1* and the bacterial pathway genes increased 2,3-butanediol yield by 2.4-fold and 11.1-fold, respectively (Figure 6). However, the yield of these strains is very low when compared with the deletion strains. The introduction of acetolactate decarboxylase pathway managed to double the yield of 2,3-butanediol in strain B2C-a1a6 to 0.089 g·g^-1^. In the engineered strain B2C-a1a5, 2,3-butanediol yield was only slightly affected by the overexpression of endogenous butanediol dehydrogenase gene. The introduction of the heterologous pathway, however, successfully increased 2,3-butanediol yield by 22% when compared with its parent strain B2C-a1a5. Despite the small enhancement of 2,3-butanediol yield, strain B2C-a1a5-ABC produced the highest titer of 2,3-butanediol, with up to 1.67 g·l^-1^, corresponding to 2,3-butanediol yield of 0.096 g·g^-1^ during microaerobic shake-flask culture.

In wild-type strain and strain B2C-a1a5, overexpression of *BDH1* rendered minimal effect on 2,3-butanediol yield. It seems likely that butanediol dehydrogenase reaction is not the bottleneck in 2,3-butanediol biosynthetic pathways in these strains. This is because the flux towards acetoin was probably not adequate in the wild-type strain expressing *BDH1*; whereas in the case of strain B2C-a1a5, it is possible that the conversion of acetaldehyde to acetoin is the rate-limiting step as significant amount of acetaldehyde accumulated in the medium (Table 3). Strain B2C-a1a6 is the only host strain that demonstrated a greater increase in 2,3-butanediol yield due the amplification of *BDH1*. It is also surprising that this strain yielded more 2,3-butanediol than ethanol (0.0887 g·g^-1^ and 0.0713 g·g^-1^, respectively) when the bacterial pathway genes are introduced. However, this strain (B2C-a1a6-ABC) only managed to consume half of the amount of glucose supplied in the fermentation medium under microaerobic condition implicating that the cell growth was greatly inhibited by certain metabolites or its cellular redox state is highly imbalanced (Data not shown). To enhance the overall flux from pyruvate to 2,3-butanediol in *S. cerevisiae*, our data in this section suggests that the overexpression of *budC or BDH1* together with *alsS* and *budA* can be an alternative to forcing carbon fluxes through the rate-limiting pyruvate decarboxylase pathway.

## Conclusions

*In silico S. cerevisiae* strain design for the production of 2,3-butanediol suggested the deletion of mitochondrial and cytosolic ADH genes. We thus constructed deletion strains with low ADH activity by the deletion of *ADH1, ADH3* and *ADH5* genes. Single deletion of major cytosolic ADH gene *ADH1* increased the production titer by around 21-fold. Detoxification mechanism of cells as a response to increased acetaldehyde pool [8], along with the failure to regenerate excess NADH efficiently through ethanol production stimulate 2,3-butanediol production in the deletion strains. Further reduction of ADH activity by additional disruption of *ADH3* or *ADH5* or both genes increased production by ~2-fold when compared to single deletion strain. But distinctively higher production was not observed, showing that the maximal conversion of acetaldehyde to acetoin and subsequently to 2,3-butanediol has been achieved by reducing ADH activity. High accumulation of acetaldehyde in these deletion strains also implies that the conversion of acetaldehyde to acetoin is rate-limiting. We have thus shown that 2,3-butanediol production can be enhanced in the engineered strains through the introduction of foreign biosynthetic pathway. Further improvement of the conversion efficiency of precursor molecules to 2,3-butanediol have to be considered in future study.

OptKnock framework has suggested a deletion strategy that led us to the hypothesis that increase in NADH, acetaldehyde and pyruvate could improve 2,3-butanediol production. This is supported by our data with strains lacking major ADH genes. Nevertheless, this hypothesis is true only to a certain extent as rational deletion strategies based on this hypothesis, including *ALD6* deletion to accumulate acetaldehyde and *GPD2* deletion to further increase NADH and pyruvate in strains with low ADH activity, did not enhance 2,3-butanediol production but increased ethanol formation instead. Problems remained to be solved to further engineer the industrial workhorse *S. cerevisiae* for 2,3-butanediol production.

In conclusion, we have demonstrated a successful implementation of *in silico* guided metabolic engineering approach for the production of a fermentative product in *S. cerevisiae.* 2,3-Butanediol producing *S. cerevisiae* strains were constructed by referring to OptKnock’s prediction. Currently, among all the engineered strains, strain B2C-a1a3a5 (*Δadh1* Δ*adh3* Δ*adh5*) achieved the highest 2,3-butanediol titer and yield under anaerobic condition (2.29 g·l^-1^ and 0.113 g·g^-1^). To our knowledge, this is the first ever report about 2,3-butanediol production in *S. cerevisiae* strain specifically engineered for its production.

## Methods

### Computational procedure

FBA-based simulation and strain design using OptKnock algorithm [18] were performed in Matlab R2010a (The MathWorks, Inc., Massachusetts, USA) with the COBRA Toolbox v2.0 [47]. Gurobi Optimizer 4.5 (Gurobi Optimization, Houston, USA) was used to performed linear programming calculations. The recently reported yeast genome-scale model iMM904 [13], consisting of 904 genes, 1577 reactions and 1228 metabolites, was used. All simulations were performed with the following constraints: oxygen uptake rate, 2mmol·gDCW^-1^·hr^-1^; glucose uptake, 10mmol·gDCW^-1^·hr^-1^; ATP required for maintenance, 1mmol·gDCW^-1^·hr^-1^. We excluded all exchange and transports reactions from the target knockouts.

### Strains, plasmid and growth conditions

All engineered strains used in this study are derived from *S. cerevisiae* BY4742 strain family (BY4742 *MATα his3Δ1 leu2Δ0 lys2Δ0 ura3Δ0*). During constructions, strains were grown and maintained on complex (YPD) medium consisting of 1% (w/v) bacto yeast extract and 2% (w/v) yeast bacto peptone supplemented with 20 g·l^-1^ glucose as carbon source. Transformants and strains harboring recombinant plasmid(s) were grown on synthetic complete dropout (SC) medium consisted of 20 g·l^-1^ glucose, 6.7 g·l^-1^ yeast nitrogen base without amino acid (Difco, BD Biosciences) and the appropriate amino acid supplement mixture (either CSM, CSM-URA, CSM-HIS, CSM-LEU or CSM-HIS-URA, MP Biomedicals). In all cases, glucose was autoclaved separately. *Escherichia coli* DH5α strain was used for amplification of plasmids. Transformed *E. coli* DH5α cells were grown at 37°C in Luria-Bertani (LB) medium containing 100ug·ml^-1^ of ampicillin.

### Construction of *S. cerevisiae* deletion strains

All the strains used in this work are listed in Table 2. Gene deletion of *S. cerevisiae* was performed by short flanking homology PCR method with Cre/loxP system for recycling of selection marker gene. pCNKanMX6, a plasmid containing *loxP-kanMX-loxP* module, were constructed from pFA6a-KanMX6 according to [48]. pTKURA3 were constructed by replacing the KanMX6 cassette with *S. cerevisiae URA3* gene including 200 nucleotides upstream and +77 nucleotides downstream of *URA3* gene. *ADH1* gene was deleted by introduction of linearized gene deletion cassette consisting of loxP-URA3-loxP sequence flanked by the 45 nucleotides upstream and downstream of *ADH1* gene to BY4742 wild-type strain. *ADH3ADH5* or *ALD6* genes were then deleted in Δ*adh1* background using the same method to construct double gene deletion strains B2C-a1a3, B2C-a1a5 and B2C-a1a6. To construct the triple deletion strains B2C-a1a3a6 and B2C-a1a5a6, *ADH3* and *ADH5* were again deleted separately from Δ*adh1* Δ*ald6* background with the previously described method. Meanwhile, strain B2C-a1a3a5 was constructed by the deletion of *ADH3* from strain B2C-a1a5. Strain B2C-a1a6g2 was constructed by the deletion of *GPD2* gene from strain B2C-a1a6. Further deletion of *ADH3* and *ADH5* genes from strain B2C-a1a6g2 resulted in the quintuple deletion strain B2C-a1a6g2a3a5. All strains were verified by colony polymerase chain reaction (PCR) with appropriate confirmation primers (Additional file 2). The aragose gel images of all gene deletion strains are provided (Additional file 3).

### Plasmid construction and transformation

The plasmids used in this study are listed in Table 4. *S. cerevisiae BDH1* gene was amplified from the genomic DNA of BY4742 strain with the primers BDH1-F-BamHI and BDH1-R-XhoI. The fragment amplified was ligated to p423GPD and p423TEF that were digested with BamHI and XhoI. The resulting construct is p423GPD-BDH1 and p423TEF-BDH1. *E. aerogenes budC* gene was amplified from the genomic DNA of *E. aerogenes* KCTC 2190 strain by PCR using the primers E.a.budC-F-BamHI and E.a.budC-R-EcoRV. *budC* gene fragment was then inserted between the BamHI-EcoRV sites of p423GPD, resulting in p423GPD-E.a.budC.

The *GAL1*/*GAL10* promoter regions of pESC-URA(Stratagene) were removed and replaced with the *PGK1*/*TEF1* divergent promoters by two-step cloning. *PGK1* promoter sequence was amplified from the genomic DNA of *S. cerevisiae* BY4742 wild-type strain with primers PGK1p-F-AatII-NotI and PGK1p-R-BamHI. It was then digested with BamHI and NotI and cloned into pESC-URA to produce pCP-URA. *TEF1* promoter region was amplified from p425TEF with primers TEF1p-R-NotI and TEF1p-F-AatII. The *TEF1* promoter fragment and pCP-URA were digested by AatII and NotI and then ligated to each other. The new plasmid generated is pCTP-URA. *Bacillus subtilis alsS* gene was amplified from the genomic DNA of *B. subtilis* using primers B.s.alsS-F-NotI and B.s.alsS-R-BglII. Meanwhile, *E. aerogenes budA* was amplified from the genomic DNA of *E. aerogenes* KCTC 2190 strain with primers E.a.budA-F-BamHI and E.a.budA-R-XhoI. *B. subtilis alsS* and *E. aerogenes budA* fragment were cloned into pCTP-URA under the control of *TEF1* promoter and *PGK1* promoter, respectively.

In all cases, PCR was performed using TaKaRa LA Taq Polymerase with GC Buffer (Takara Bio Inc, Shiga, Japan). Yeast strains were transformed using the lithium acetate/PEG/SS-DNA method [49]. After transformation, successful transformants were selected on SC media without the appropriate amino acids.

### Batch fermentations

For all fermentations, pre-cultures were prepared by inoculating 5 ml of SC medium in a 50 ml screw-capped tube with a fresh colony grown on solid medium. Batch fermentations were performed in 250 ml Erlenmeyer flasks containing 50 ml of synthetic complete dropout medium. Microaerobic condition was maintained in the flasks by sealing with rubber stoppers. For anaerobic fermentations, 50 ml of SC medium supplemented with 0.42 g·l^-1^ Tween 80 and 0.01 g·l^-1^ cholesterol in a 150 ml serum bottle was purged with pure nitrogen gas for 15 minutes to establish anaerobic condition. Initial OD_660nm_ was adjusted at 0.05. Fermentations were performed in shaking incubator at 30°C, with agitation at 250 rpm.

### Analytical methods

Cell growth was determined by measuring the absorbance at 660 nm using UV–VIS spectrophotometer (Shimazu UV mini 1240, Tokyo, Japan). In addition, we determined the dry cell mass of yeast at the end of shake-flask cultivation by filtering 30 ml of culture through pre-weighed cellulose acetate membrane filters (Chmlab Group, Barcelona, Spain) with a pore size of 0.45 μm. Filters were dried at 65°C for 48 hours and weighed again. Then, we calculated the dry cell mass and estimated the cell biomass by using the dry cell mass/OD660 ratio at the end of cultivation.

For extracellular metabolite analysis, 1 ml of culture samples were collected at appropriate time point. Isopropyl alcohol was added to the 1 ml-sample as internal standard and vortexed for 1 min. The samples were then centrifuged (13500 rpm, 5 min) and 200 μl of supernatants were transferred to HPLC autosampler vials. Extracellular concentrations of glucose, glycerol, ethanol, acetate, succinate, acetoin and 2,3-butanediol were determined with a high performance liquid chromatography (HPLC) system (ACME-9000, Younglin Instrument, Seoul, South Korea) equipped with a refractive index detector (RID). Analytes were separated using Sugar SH1011 column (Shodex, Tokyo, Japan) with 10 mM sulfuric acid as mobile phase pumped at 0.5ml·min^-1^. The column and the detector were set to 75°C and 45°C, respectively. The order of elution was glucose, succinic acid, glycerol, acetate, acetoin, 2,3-butanediol, ethanol and isopropyl alcohol. For quantitative analysis, standards for all metabolite at five different concentration levels were prepared. A calibration curve for each component was made and from these calibration curves concentrations of metabolites were calculated. The standard for 2,3-butanediol (≥99.0% (GC), Fluka Analytical, Sigma-Aldrich) and the extracellular 2,3-butanediol from the fermentation samples are a mixture of racemic and meso forms. Extracellular acetaldehyde concentrations were determined with Megazyme Acetaldehyde Assay Kit (Megazyme International Ireland, Wicklow, Ireland) according to the manufacturer’s instructions.

## Abbrevation list

FBA: flux balance analysis; PDH: pyruvate dehydrogenase; ADH: alcohol dehydrogenase; G3P: glycerol-3-phosphate.

## Competing interests

The authors declare that they have no competing interests.

## Authors’ contributions

MKO and JL conceived the study. MKO designed and supervised the study, and contributed to manuscript writing. CYN participated in the design of the study, performed the experiments and drafted the manuscript. MYJ helped to conduct HPLC analysis. All authors read and approved the final manuscript.

## Supplementary Material

Additional file 1 **Additional description for Table **1**.** (a) A list of the corresponding enzymes, reaction equations and subsystems for target reactions listed in Table 1 and (b) a figure showing the 2,3-butanediol production envelope of OptKnock strain A. Click here for file

Additional file 2 **Oligonucleotides used in this study.** A list of oligonucleotides that were used in this study. Click here for file

Additional file 3 **Verification of gene deletion strains by colony PCR.** The PCR profiles of wild-type and gene deletion strains. Click here for file

## References

[B1] Ji X-J Huang H Ouyang P-K Microbial 2,3-butanediol production: A state-of-the-art review Biotechnol Adv 2011 29 351 364 10.1016/j.biotechadv.2011.01.007 21272631

[B2] Celinska E Grajek W Biotechnological production of 2,3-butanediol–current state and prospects Biotechnol Adv 2009 27 715 725 10.1016/j.biotechadv.2009.05.002 19442714

[B3] Yang T Rao Z Zhang X Lin Q Xia H Xu Z Yang S Production of 2,3-butanediol from glucose by GRAS microorganism Bacillus amyloliquefaciens J Basic Microbiol 2011 51 650 658 10.1002/jobm.201100033 21780143

[B4] Chen GC Jordan F Brewers' yeast pyruvate decarboxylase produces acetoin from acetaldehyde: a novel tool to study the mechanism of steps subsequent to carbon dioxide loss Biochemistry 1984 23 3576 3582 10.1021/bi00311a002 6383467

[B5] Sergienko EA Jordan F Catalytic acid–base groups in yeast pyruvate decarboxylase. 2. Insights into the specific roles of D28 and E477 from the rates and stereospecificity of formation of carboligase side products Biochemistry 2001 40 7369 7381 10.1021/bi002856m 11412091

[B6] Blomqvist K Suihko ML Knowles J Penttila M Chromosomal Integration and Expression of Two Bacterial alpha-Acetolactate Decarboxylase Genes in Brewer's Yeast Appl Environ Microbiol 1991 57 2796 2803 16348559 10.1128/aem.57.10.2796-2803.1991 PMC183877

[B7] Suihko ML Blomqvist K Penttila M Gisler R Knowles J Recombinant brewer's yeast strains suitable for accelerated brewing J Biotechnol 1990 14 285 300 10.1016/0168-1656(90)90113-P 1366907

[B8] Remize F Roustan J Sablayrolles J Barre P Dequin S Glycerol overproduction by engineered Saccharomyces cerevisiae wine yeast strains leads to substantial changes in by-product formation and to a stimulation of fermentation rate in stationary phase Appl Environ Microbiol 1999 65 143 9872772 10.1128/aem.65.1.143-149.1999 PMC90995

[B9] Ehsani M Fernandez MR Biosca JA Julien A Dequin S Engineering of 2,3-butanediol dehydrogenase to reduce acetoin formation by glycerol-overproducing, low-alcohol Saccharomyces cerevisiae Appl Environ Microbiol 2009 75 3196 3205 10.1128/AEM.02157-08 19329666 PMC2681661

[B10] Romano P Suzzi G Origin and production of acetoin during wine yeast fermentation Appl Environ Microbiol 1996 62 309 16535224 10.1128/aem.62.2.309-315.1996 PMC1388762

[B11] Lewis NE Nagarajan H Palsson BO Constraining the metabolic genotype–phenotype relationship using a phylogeny of in silico methods Nat Rev Microbiol 2012 10 291 305 22367118 10.1038/nrmicro2737 PMC3536058

[B12] Forster J Famili I Fu P Palsson BO Nielsen J Genome-scale reconstruction of the Saccharomyces cerevisiae metabolic network Genome Res 2003 13 244 253 10.1101/gr.234503 12566402 PMC420374

[B13] Mo ML Palsson BO Herrgard MJ Connecting extracellular metabolomic measurements to intracellular flux states in yeast BMC Syst Biol 2009 3 37 10.1186/1752-0509-3-37 19321003 PMC2679711

[B14] Herrgard MJ Swainston N Dobson P Dunn WB Arga KY Arvas M Bluthgen N Borger S Costenoble R Heinemann M A consensus yeast metabolic network reconstruction obtained from a community approach to systems biology Nat Biotechnol 2008 26 1155 1160 10.1038/nbt1492 18846089 PMC4018421

[B15] Duarte NC Herrgard MJ Palsson BO Reconstruction and validation of Saccharomyces cerevisiae iND750, a fully compartmentalized genome-scale metabolic model Genome Res 2004 14 1298 1309 10.1101/gr.2250904 15197165 PMC442145

[B16] Orth JD Thiele I Palsson BO What is flux balance analysis? Nat Biotechnol 2010 28 245 248 10.1038/nbt.1614 20212490 PMC3108565

[B17] Edwards JS Covert M Palsson B Metabolic modelling of microbes: the flux-balance approach Environ Microbiol 2002 4 133 140 10.1046/j.1462-2920.2002.00282.x 12000313

[B18] Burgard AP Pharkya P Maranas CD Optknock: a bilevel programming framework for identifying gene knockout strategies for microbial strain optimization Biotechnol Bioeng 2003 84 647 657 10.1002/bit.10803 14595777

[B19] Fong SS Burgard AP Herring CD Knight EM Blattner FR Maranas CD Palsson BO In silico design and adaptive evolution of Escherichia coli for production of lactic acid Biotechnol Bioeng 2005 91 643 648 10.1002/bit.20542 15962337

[B20] Xu P Ranganathan S Fowler ZL Maranas CD Koffas MA Genome-scale metabolic network modeling results in minimal interventions that cooperatively force carbon flux towards malonyl-CoA Metab Eng 2011 13 578 587 10.1016/j.ymben.2011.06.008 21763447

[B21] Yim H Haselbeck R Niu W Pujol-Baxley C Burgard A Boldt J Khandurina J Trawick JD Osterhout RE Stephen R Metabolic engineering of Escherichia coli for direct production of 1,4-butanediol Nat Chem Biol 2011 7 445 452 10.1038/nchembio.580 21602812

[B22] Matsuda F Furusawa C Kondo T Ishii J Shimizu H Kondo A Engineering strategy of yeast metabolism for higher alcohol production Microb Cell Fact 2011 10 70 10.1186/1475-2859-10-70 21902829 PMC3184262

[B23] Asadollahi MA Maury J Patil KR Schalk M Clark A Nielsen J Enhancing sesquiterpene production in Saccharomyces cerevisiae through in silico driven metabolic engineering Metab Eng 2009 11 328 334 10.1016/j.ymben.2009.07.001 19619667

[B24] Schuster S Pfeiffer T Fell DA Is maximization of molar yield in metabolic networks favoured by evolution? J Theor Biol 2008 252 497 504 10.1016/j.jtbi.2007.12.008 18249414

[B25] Kennedy CJ Boyle PM Waks Z Silver PA Systems-level engineering of nonfermentative metabolism in yeast Genetics 2009 183 385 10.1534/genetics.109.105254 19564482 PMC2746161

[B26] Bro C Regenberg B Forster J Nielsen J In silico aided metabolic engineering of Saccharomyces cerevisiae for improved bioethanol production Metab Eng 2006 8 102 111 10.1016/j.ymben.2005.09.007 16289778

[B27] Pronk JT Steensma HY Van Dijken JP Pyruvate metabolism in Saccharomyces cerevisiae Yeast 1996 12 1607 1633 10.1002/(SICI)1097-0061(199612)12:16<1607::AID-YEA70>3.0.CO;2-4 9123965

[B28] Nissen TL Kielland-Brandt MC Nielsen J Villadsen J Optimization of Ethanol Production in Saccharomyces cerevisiae by Metabolic Engineering of the Ammonium Assimilation Metab Eng 2000 2 69 77 10.1006/mben.1999.0140 10935936

[B29] Bieganowski P Seidle HF Wojcik M Brenner C Synthetic lethal and biochemical analyses of NAD and NADH kinases in Saccharomyces cerevisiae establish separation of cellular functions J Biol Chem 2006 281 22439 22445 10.1074/jbc.M513919200 16760478

[B30] Drewke C Thielen J Ciriacy M Ethanol formation in adh0 mutants reveals the existence of a novel acetaldehyde-reducing activity in Saccharomyces cerevisiae J Bacteriol 1990 172 3909 2193925 10.1128/jb.172.7.3909-3917.1990 PMC213373

[B31] Dickinson JR Salgado LE Hewlins MJ The catabolism of amino acids to long chain and complex alcohols in Saccharomyces cerevisiae J Biol Chem 2003 278 8028 8034 10.1074/jbc.M211914200 12499363

[B32] Weimer EP Rao E Brendel M Molecular structure and genetic regulation of SFA, a gene responsible for resistance to formaldehyde in Saccharomyces cerevisiae, and characterization of its protein product Mol Gen Genet MGG 1993 237 351 358 10.1007/BF00279438 8483449

[B33] Smith MG Des Etages SG Snyder M Microbial Synergy via an Ethanol-Triggered Pathway Mol Cell Biol 2004 24 3874 3884 10.1128/MCB.24.9.3874-3884.2004 15082781 PMC387754

[B34] de Smidt O du Preez JC Albertyn J Molecular and physiological aspects of alcohol dehydrogenases in the ethanol metabolism of Saccharomyces cerevisiae FEMS Yeast Res 2012 12 33 47 10.1111/j.1567-1364.2011.00760.x 22094012

[B35] Skory CD Lactic acid production by Saccharomyces cerevisiae expressing a Rhizopus oryzae lactate dehydrogenase gene J Ind Microbiol Biotechnol 2003 30 22 27 12545382 10.1007/s10295-002-0004-2

[B36] Stanley GA Douglas NG Every EJ Tzanatos T Pamment NB Inhibition and stimulation of yeast growth by acetaldehyde Biotechnol Lett 1993 15 1199 1204 10.1007/BF00130297

[B37] Tokuhiro K Ishida N Nagamori E Saitoh S Onishi T Kondo A Takahashi H Double mutation of the PDC1 and ADH1 genes improves lactate production in the yeast Saccharomyces cerevisiae expressing the bovine lactate dehydrogenase gene Appl Microbiol Biotechnol 2009 82 883 890 10.1007/s00253-008-1831-5 19122995

[B38] Eglinton JM Heinrich AJ Pollnitz AP Langridge P Henschke PA de Barros Lopes M Decreasing acetic acid accumulation by a glycerol overproducing strain of Saccharomyces cerevisiae by deleting the ALD6 aldehyde dehydrogenase gene Yeast 2002 19 295 301 10.1002/yea.834 11870853

[B39] Ludovico P Sousa MJ Silva MT Leão C Côrte-Real M Saccharomyces cerevisiae commits to a programmed cell death process in response to acetic acid Microbiology 2001 147 2409 2415 11535781 10.1099/00221287-147-9-2409

[B40] Roustan J Sablayrolles J Impact of the addition of electron acceptors on the by-products of alcoholic fermentation Enzym Microb Technol 2002 31 142 152 10.1016/S0141-0229(02)00086-8

[B41] Ansell R Granath K Hohmann S Thevelein JM Adler L The two isoenzymes for yeast NAD + −dependent glycerol 3-phosphate dehydrogenase encoded by GPD1 and GPD2 have distinct roles in osmoadaptation and redox regulation EMBO J 1997 16 2179 2187 10.1093/emboj/16.9.2179 9171333 PMC1169820

[B42] Gollop N Damri B Chipman D Barak Z Physiological implications of the substrate specificities of acetohydroxy acid synthases from varied organisms J Bacteriol 1990 172 3444 3449 2345154 10.1128/jb.172.6.3444-3449.1990 PMC209156

[B43] Carballo J Martin R Bernardo A Gonzalez J Purification, characterization and some properties of diacetyl(acetoin) reductase from Enterobacter aerogenes Eur J Biochem 1991 198 327 332 10.1111/j.1432-1033.1991.tb16019.x 2040298

[B44] Ehsani M Fernandez MR Biosca JA Dequin S Reversal of coenzyme specificity of 2,3-butanediol dehydrogenase from Saccharomyces cerevisae and in vivo functional analysis Biotechnol Bioeng 2009 104 381 389 10.1002/bit.22391 19507198

[B45] Wach A PCR-synthesis of marker cassettes with long flanking homology regions for gene disruptions in S. cerevisiae Yeast 1996 12 259 265 10.1002/(SICI)1097-0061(19960315)12:3<259::AID-YEA901>3.0.CO;2-C 8904338

[B46] Mumberg D Müller R Funk M Yeast vectors for the controlled expression of heterologous proteins in different genetic backgrounds Gene 1995 156 119 122 10.1016/0378-1119(95)00037-7 7737504

[B47] Schellenberger J Que R Fleming RM Thiele I Orth JD Feist AM Zielinski DC Bordbar A Lewis NE Rahmanian S Quantitative prediction of cellular metabolism with constraint-based models: the COBRA Toolbox v2.0 Nat Protoc 2011 6 1290 1307 10.1038/nprot.2011.308 21886097 PMC3319681

[B48] Güldener U Heck S Fiedler T Beinhauer J Hegemann JH A new efficient gene disruption cassette for repeated use in budding yeast Nucleic Acids Res 1996 24 2519 10.1093/nar/24.13.2519 8692690 PMC145975

[B49] Gietz RD Schiestl RH Willems AR Woods RA Studies on the transformation of intact yeast cells by the LiAc/SS‒DNA/PEG procedure Yeast 1995 11 355 360 10.1002/yea.320110408 7785336

