# Supplementary material for: Production of 2,3-butanediol in Saccharomyces cerevisiae by in silico aided metabolic engineering
Source: Microb Cell Fact. 2012 May 28;11:68. doi: 10.1186/1475-2859-11-68 (PMC3442981; doi:10.1186/1475-2859-11-68)
Supplement: Additional file 1 — Additional description for Table 1. (a) A list of the corresponding enzymes, reaction equations and subsystems for target reactions listed in Table 1 and (b) a figure showing the 2,3-butanediol production envelope of OptKnock strain A. [file 1475-2859-11-68-S1.pdf]

Additional file 1. Additional description for Table 1

(a) The corresponding enzymes, reaction equation and subsystem for target reactions listed in Table 1.

Reaction abbreviation	Enzyme	Reaction equation	Subsystem
ALCD2ir	Alcohol dehydrogenase (cytosol)	ACALD + H^+ + NADH → ETOH+ NAD	Pyruvate metabolism
ALCD2irm	Alcohol dehydrogenase (mitochondrial)	ACALD + H^+ + NADH → ETOH + NAD	Pyruvate metabolism
ALCD2x	Alcohol dehydrogenase	ETOH + NAD ↔ ACALD + H^+ + NADH	Pyruvate metabolism
ASPTA	Aspartate transaminase	AKG + ASP-L ↔ GLU-L + OAA	Alanine and aspartate metabolism
CYTK1	Cytidylate kinase	ATP + CMP ↔ ADP + CDP	Nucleotide salvage pathway
GLUDyi	Glutamate dehydrogenase	AKG + H + NADPH + NH4 → GLU-L + H_2O + NADP	Glutamate metabolism
GTPCI	GTP cyclohydrolase I	GTP + H_2O → AHDT + FOR + H^+	Folate metabolism
MDH	Malate dehydrogenase	MAL-L + NAD ↔ H^+ + NADH + OAA	Oxidative phosphorylation
NADK	NAD kinase	ATP + NAD → ADP + H^+ + NADP	NAD biosynthesis
NDPK3	Nucleoside-diphosphate kinase	ATP + CDP ↔ ADP + CTP	Nucleotide salvage pathway
PDHm	Pyruvate dehydrogenase	COA + NAD + PYR → ACCOA + CO_2 + NADH	Glycolysis/Gluconeoge nesis
TMDPP	Thymidine phosphorylase	PI + THYMD ↔ 2DR1P + THYM	Purine and pyrimidine biosynthesis

(b)

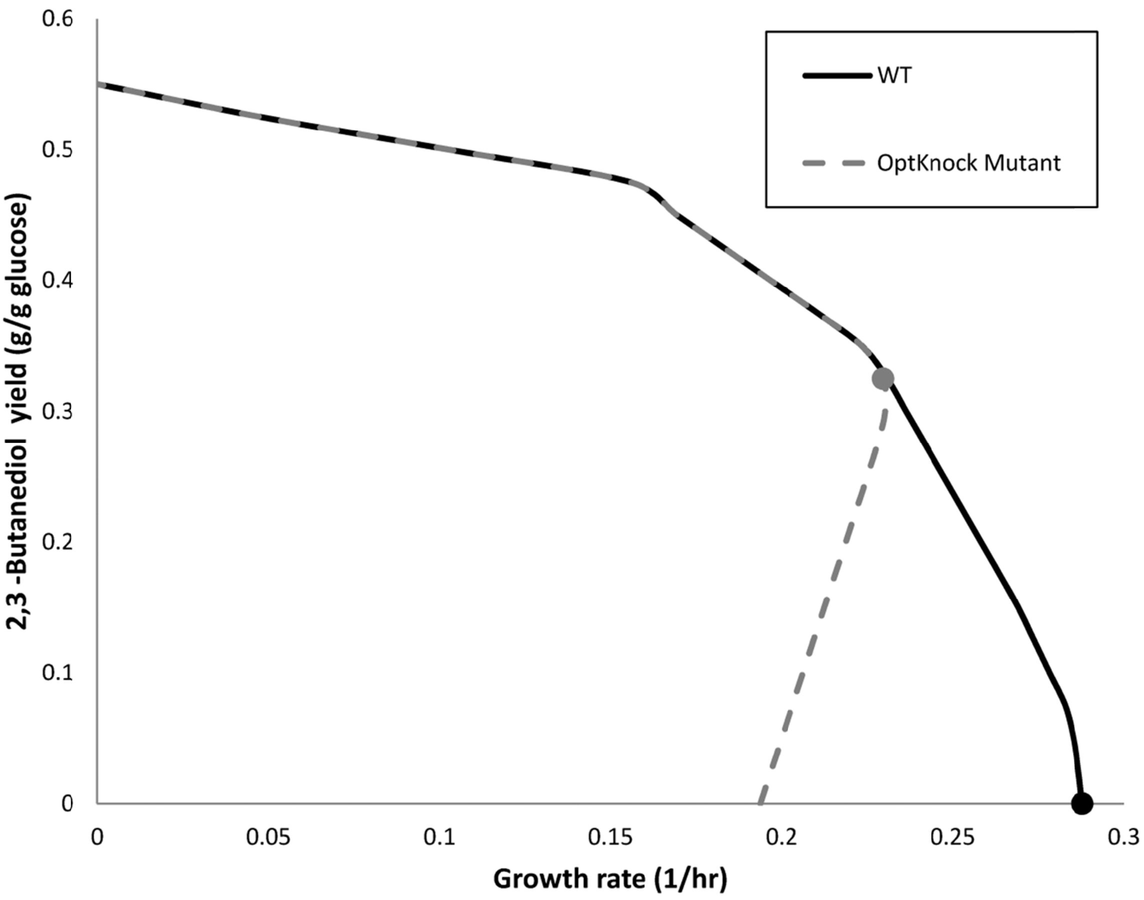

2,3-Butanediol production envelope of OptKnock strain A in Table 1. Plot shows maximum attainable 2,3-butanediol yield as a function of the maximum biomass production rate. Grey and black circles represent FBA optimal point of OptKnock mutant (Δ*adh1* Δ*adh3* Δ*adh4* Δ*adh5* Δ*sfa1*) and reference strain respectively. Oxygen uptake rate of 2 mmol·gDCW^{-1}.hr^{-1}, glucose uptake of 10 mmol·gDCW^{-1}.hr^{-1} and ATP required for maintenance of 1 mmol·gDCW^{-1}.hr^{-1}are assumed.
