# Supplementary material for: Production of 2,3-butanediol in Saccharomyces cerevisiae by in silico aided metabolic engineering
Source: Microb Cell Fact. 2012 May 28;11:68. doi: 10.1186/1475-2859-11-68 (PMC3442981; doi:10.1186/1475-2859-11-68)
Supplement: Additional file 2 — Oligonucleotides used in this study. A list of oligonucleotides that were used in this study. [file 1475-2859-11-68-S2.pdf]

Additional file 2. Oligonucleotides used in this study

Oligonucleotide	Sequence (5' to 3')
ADH1-For-pUG	TTTCAAGCTATACCAAGCATACAATCAACTATCTCATATACAATGCGTACG CTGCAGGTCGAC
ADH1-Rev-pUG	AACTTATTTAATAATAAAAATCATAAATCATAAGAAATTCGCTTAACTAGT GGATCTGATATC
ADH1-A-conf	CTTCATTCACGCACACTACTCTCTA
ADH1-B-conf	ACTAATGGTAGCTTAACTGGCAATG
ADH1-C-conf	CTACAAGGCTTTGAAGTCTGCTAAC
ADH1-D-conf	GGACATAAAATACACACCGAGATTC
URA3-B-conf	GACCTAATGCTTCAACTAACTCCAG
URA3-C-conf	ACATTGCGAAGAGCGACAAA
ADH3-For-pUG	CAGTTAAAACTAGGAATAGTATAGTCATAAGTTAACACCATCATG CGTACGCTGCAGGTCGAC
ADH3-Rev-pUG	AAACAAAGACTTTCATAAAAAGTTTGGGTGCGTAACACGCTATTA GTGGATCTGATATCACCT
ADH3-A-conf	GTCCGTACACTGTCCTTTTGTTACT
ADH3-B-conf	AACCTAGTTTGACAACTACACCAGC
ADH3-C-conf	GCCTTAGACTTCTTTAGCAGAGGTT
ADH3-D-conf	GAGGCTGATTATTAAGAACACAGGA
ADH5-For-pUG	TAAGAAAATTATTTAACTACATATCTACAAAATCAAAGCATCATGCGTACG CTGCAGGTCGAC
ADH5-Rev-pUG	TATAAAAAGTAAAAATATATTCATCAAATTCGTTACAAAAGATCAGTGGAT CTGATATCACCT
ADH5-A-conf	TGTTTGTTCTGCTATCTGCTTGTAG
ADH5-B-conf	GTACCATCCAAATAAGGACATTGAG
ADH5-C-conf	TGTGTTGGAAATAGAGCTGATACAA
ADH5-D-conf	ATTAAATTTCTTTTCCGTAAATGGC
ALD6-For-pUG	GAAACATCTTTAACATACACAAACACATACTATCAGAATACAATGCGTACG CTGCAGGTCGAC
ALD6-Rev-pUG	AGTATTTTGTGTATATGACGGAAAGAAATGCAGGTTGGTACATTAACTAGT GGATCTGATATC

ALD6-A-conf	CATCCAGCTTCTATATCGCTTTAAC
ALD6-B-conf	AGCAGCATCTCTTAGACAGTTGATT
ALD6-C-conf	TAGTAAACGGTATTTTCAAGAACGC
ALD6-D-conf	AATATGATTTTGTGTGGGATGTTTT
PGK1p-F-AatII-NotI	GTTTGCGGCCGCCCGACGTCGGAAGTACCT
GPD2-For-pUG	TTCTCTTTCCCTTTCCTTTTCCTTCGCTCCCCTTCCTTATCAATG CGTACGCTGCAGGTCGAC
GPD2-Rev-pUG	AGGCAACAGGAAAGATCAGAGGGGGAGGGGGGGGGAGAGTGTCTAACTA GTGGATCTGATATC
GPD2-A-conf	CTCTGCCATTGTTATATTACGCTTT
GPD2-B-conf	GTTACCAGAACCAATCACTGTAACC
GPD2-C-conf	GGCTTACCAACTACCAAAGGATTAT
GPD2-D-conf	GAGAATTACTGCAGTGAAAAAGCTC
PGK1p-R-BamHI	GCCGGATCCTTGTTTTATATTTGTTGTAAAAAGTAG
TEF1p-F-AatII	GGGATCCTTGGACGTCCACACACCATAGCTTCAA
TEF1p-R-NotI	GTTGTTGCGGCCGCTTGTAATTAAAACTTAGATTAGATTGC
B.s.alsS-F-NotI	GGTAGCGGCCGCATGTTGACAAAAGCAACA
B.s.alsS-R-BglII	CTCAAGATCTTGATGCGCAGAGTGCTA
E.a.budA-F-BamHI	GTTGGATCC TGGAACTGTGAGCTGAATCG
E.a.budA-R-XhoI	CAA CTCGAGCTGCGGATACTGTTTGTCCA
E.a.budC-F-BamHI	CCAGGATCCATGAAAAAAGTCGCA
E.a.budC-R-EcoRV	GGATATCTTAATTGAATACCATCCC
BDH1-F-BamHI	AAA GGATCC ATGAGAGCTTTGGC
BDH1-R-XhoI	GGG CTCGAG TTACTTCAT TTCACC
