# Supplementary material for: Production of 2,3-butanediol in Saccharomyces cerevisiae by in silico aided metabolic engineering
Source: Microb Cell Fact. 2012 May 28;11:68. doi: 10.1186/1475-2859-11-68 (PMC3442981; doi:10.1186/1475-2859-11-68)
Supplement: Additional file 3 — Verification of gene deletion strains by colony PCR. The PCR profiles of wild-type and gene deletion strains. [file 1475-2859-11-68-S3.pdf]

Additional file 3. Verification of gene deletion strains by colony PCR

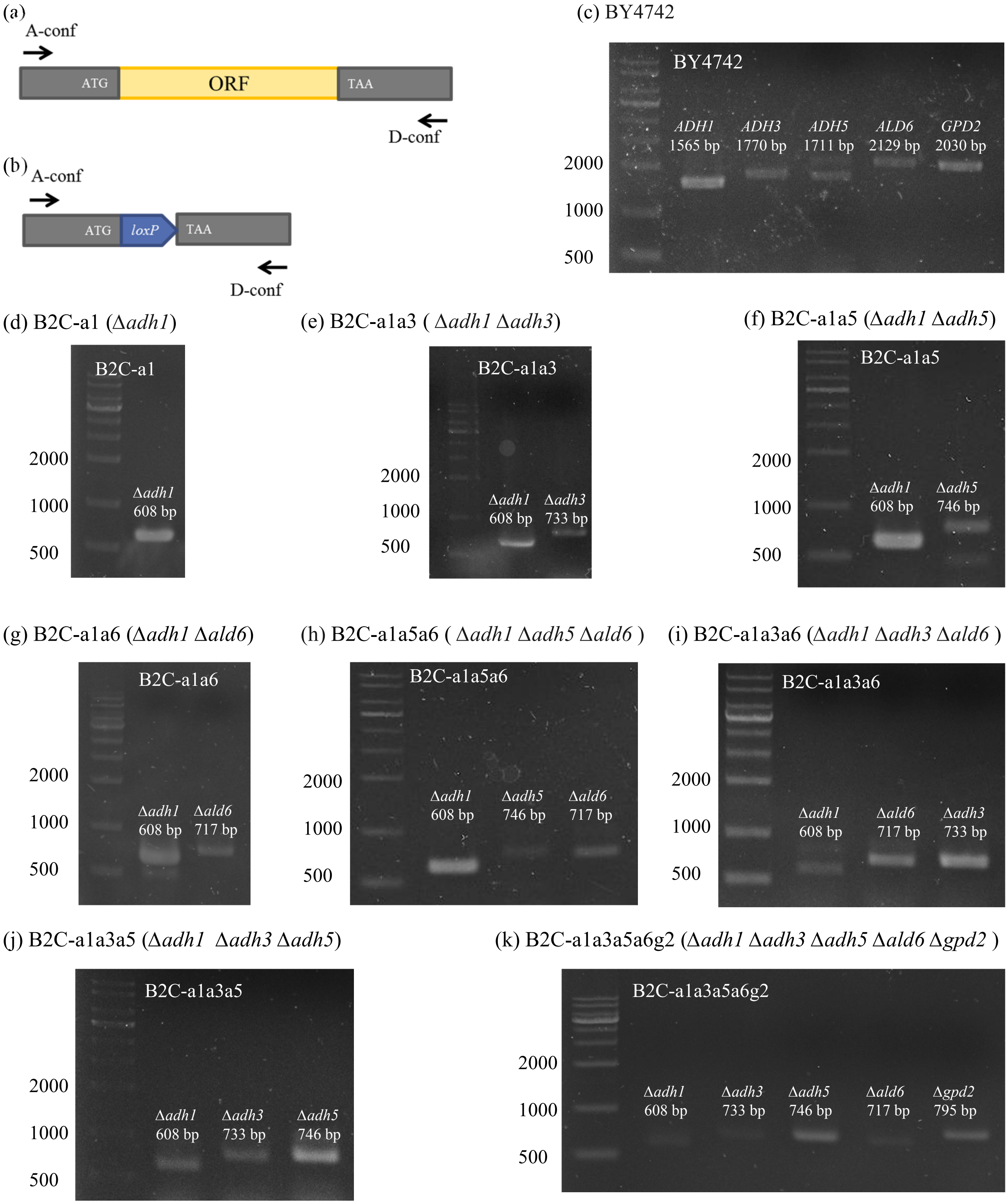

Additional file 2. The PCR profiles of wild type and mutant strains. (a), (c) Amplification of intact genes from wild-type strain with primer A-conf and D-conf (additional file 1) of the respected genes gave a larger PCR product than the deletion strain: *ADH1* (1565 bp), *ADH3* (1770 bp), *ADH5* (1711 bp), *ALD6* (2129 bp) and *GPD2* (2030 bp). (b), (d-i) The PCR profiles of the gene deletion strains. As only a *loxP* sequence remains in the locus of the deleted gene, the same primer pair generates a shorter PCR product: *adh1* (608 bp), *adh3* (733 bp), *adh5* (746 bp), *ald6* (717 bp) and *gpd2* (795 bp). The first lane in each gel represents the 1Kb DNA Ladder Marker (Elpis Biotech Inc, South Korea).
